# Precise Marketing of E-Commerce Products Based on KNN Algorithm

**DOI:** 10.1155/2022/4966439

**Published:** 2022-08-11

**Authors:** Jianfeng Zou, Hui Li

**Affiliations:** Guangzhou College of Technology and Business, Guangzhou, China

## Abstract

In order to better understand the purchase decision-making process of consumers, this paper makes an in-depth study on the precision marketing of e-commerce products on the basis of KNN algorithm. Through data mining, classic KNN algorithm, BPNN algorithm, and other methods, this paper takes the price and purchase intention of e-commerce agricultural products as an example. Based on the classic nearest neighbor algorithm, binomial function is combined with Euclidean distance formula when calculating the nearest neighbor through similarity. The particle swarm optimization algorithm is used to optimize the binomial function coefficient and the *K* value of the nearest neighbor algorithm, and the results of the best prediction model for the prediction application of e-commerce agricultural product price and purchase intention are established. Both pricing strategies and promotion strategies will weaken the compromise effect of consumers when they choose e-commerce agricultural products. After studying the calculation method of the KNN algorithm, it not only correctly predicts the price of e-commerce agricultural products but also makes a corresponding prediction and analysis of consumers' purchase intention of e-commerce agricultural products, with the highest accuracy of 94.2%. At the same time, in the future precision marketing process, e-commerce agricultural products enterprises use data technology to achieve precision marketing, which effectively changes the shortcomings of traditional marketing and improves the product marketing effect and economic benefits.

## 1. Introduction

Compared with traditional marketing methods, precision marketing has two advantages: one is accuracy. General precision marketing activities are based on mining and analyzing consumer big data, so they can ensure the accuracy of marketing. Second, real-time E-commerce enterprises can observe the changes of consumer preferences in real time and adjust the marketing plan under this premise and accurately push marketing messages related to products for users. Marketing activities can greatly improve the response speed to users' subjective and nondetermined factors. This requires accurate prediction and control of the prices of e-commerce products and then recommend products with different prices to consumers according to their acceptance of the prices of products. The information revolution has brought more efficient production, storage and consumption of data, as well as the explosive growth of data and then people find that the original use of data has caused a huge waste. People reached a consensus and began to analyze the data systematically, which gave birth to data mining technology and brought huge social benefits. After e-commerce platforms have become an indispensable part of modern life, consumers' consumption habits have gradually changed, including the daily consumption and consumption of agricultural products. For example, agricultural products have gradually become a hot part of online e-commerce platforms. China is a big country in agricultural production, and agriculture is still the basic industry of China. Especially in the context of China's large population and low urbanization popularization, the reasonable planting, production and sales of agricultural products, and the stability of agricultural products prices are closely related to the daily life of every citizen. However, in recent years, in terms of production, there has been a phenomenon of low purchase price of dishes and low planting willingness of farmers. And, in the sales stage, the prices of e-commerce agricultural products fluctuate frequently, and consumers directly call out that cannot afford to consume the news frequently appeared. These two extremes occur frequently and play out alternately, even making agricultural products more expensive than meat and eggs and other byproducts and seriously affecting the normal life of resident. E-commerce enterprises that implement precision marketing can save marketing advertising costs and transaction costs based on these two advantages and maximize the value of marketing activities. At the same time, with the help of precision marketing activities, potential consumers will be transformed into actual consumer groups, and the speed of users making purchase decisions and behavior will be improved to further improve their own economic benefits. The price of agricultural products is affected by macroeconomic supply and demand fluctuation, seasonal climate, and other aspects, and generally, the market and government play the main regulating role. In order to improve the degree of marketization of agricultural products, it is necessary to vigorously develop agricultural product futures trading, which in turn requires the government and the management departments of various industries to streamline administration and delegate power and let the market resolve market matters. At present, some industry management departments have cancelled the “rules that state-owned enterprises are not allowed to participate in futures trading.” Some departments have begun to encourage grain companies to use the futures market to avoid business risks, and more companies in the agricultural product industry chain are willing to use futures tools to enterprise production and operation services. Especially under China's national conditions and economic system, with the development of agricultural information industry, government departments have the most direct data in agricultural monitoring, statistics, market analysis, management, information services, production and management, and the market of origin of agricultural products. In addition, with the rapid development of computer hardware and software, computer science and technology, the continuous improvement of agricultural information management system, and the mature application of various powerful databases, these data can be accurately and completely preserved. Researchers in sociology, economics, management, and other fields have extensively discussed and analyzed these social phenomena and statistical data and put forward solutions in their respective fields. In computer science and technology, data mining is a subject that can mine valuable knowledge from a large number of disordered data. Using data mining technology, a reasonable agricultural product application model was established to analyze the relationship between the fluctuation of agricultural product price and the trend of price index growth. After analyzing the relationship between price and product market, we can use visualization technology to display and finally realize the guidance of agricultural product planting and breeding, stabilize the price change, reduce the CPI growth trend, and solve the complex and huge contradiction between farmers and consumers, which has important practical significance. All marketing activities should be based on a full understanding of customer data and information, so as to achieve precision marketing. Before implementing precision marketing, e-commerce enterprises should fully understand the basic information of customers, customer consumption behavior, customer comprehensive data analysis, customer behavior data, etc., and understand customers' purchasing power, purchase preferences, and loyalty to products and enterprises. In particular, the establishment of agricultural, strong stability, and low computational complexity can realize the accurate prediction of agricultural product price fluctuations, which plays an important guiding role in the rational planting, production, and sales of agricultural products. From the perspective of computer social and development, the collected data are applied and analyzed. On the one hand, it can promote the application of data model in agricultural product marketing. On the other hand, the precision marketing of agricultural products in e-commerce platform is discussed.

## 2. Literature Review

Zardari and others have studied KNN algorithm. At the same time, they think that data mining, as an interdisciplinary subject, is one of the most advanced research directions of data workers in the world. It has a broad application prospect and has attracted the attention of researchers in many fields at home and abroad [1]. Ji and others researched data mining, which is also known as knowledge discovery in data (KDD). The word first appeared in a panel discussion of the 11th International Joint Conference on Artificial Intelligence held in Detroit in August 1989, and the content of the conference was published in 1991 [2]. Zhu and others expressed that ACM Data Mining and Knowledge Discovery Committee was established and ACM SIGKDD Conference on Knowledge Discovery and Data Mining (KDD) was organized. KDD has developed into a top international conference in the field of data mining [3]. The SIGKDD conference is held once a year and has been held for 19 sessions, including 2012 in Beijing, China, which is the only conference held in Asian countries. In August 2014, the 20th KDD conference was held in New York, USA. In addition, Xie and others held that the international Conference on Neural Information Processing Systems related to data Mining, the International Conference of the Association for Computational Linguistics, the International Conference on Knowledge Representation and Reasoning Theory, and the International Conference on Industrial Data Mining are among the top international conferences in this field [4]. Wei and other scholars' foreign research on data mining technology started earlier, and the theoretical basis and interdisciplinary research is in a leading position. Driven by the commercial interest of scientific research, financial telecommunication, retail and medical industries, a large number of mature data mining products have been produced. There are a large number of foreign institutions and universities conducting data mining research [5]. Yilmaz held that, with the advent of the era of big data, there are many start-up companies based on big data processing and business intelligence analysis. Compared with foreign countries, Chinese data mining research started a little later, and the research direction is more focused on practical application, good at using the latest technology to solve practical problems. Chinese scholars are at the forefront of the international level in solving specific application research and have published a large number of papers with academic impact in the fields of classification model unsupervised learning and social network [6], but there is still a lack of groundbreaking achievements in defining brand new scientific problems, such as the infrastructure of big data. Since various data mining tools are designed with different algorithms, they work in different ways. Therefore, choosing the right data mining tool is a very difficult task. Ma, Du, etc., held that, in terms of basic research, there is still a certain gap between Chinese scholars and international leading research teams, but with the development of the information age, the gap is narrowing. Chinese research on data mining is mainly concentrated in universities and research institutes. The famous Institute of Machine Learning and Data Mining (LAMDA) of Nanjing University is mainly engaged in the research work of artificial intelligence machine learning data mining pattern recognition and other fields [7]. Farhadi Esmaeily and others held that researchers at Renmin University of China have in-depth research on key technologies of analytic-oriented high-performance databases. The Nankai-Baidu joint Laboratory established by Nankai University and Baidu has carried out key research in the management technology of parallel and distributed algorithms for massive data. In addition, Tsinghua University Knowledge Engineering Laboratory, software research institute, Department of computer science, Xiamen University data mining center, China University of social development, China University of Science and Technology, and other universities have conducted in-depth research on data mining. Many practical algorithms have been proposed by the scientific and technological workers in these universities and research institutes. Moreover, the advantages of high-performance computing such as GPU acceleration, parallel computing, distributed computing, and cloud computing platform are reflected in data mining technology, which actively promotes the development of data mining in China [8]. Zhou and others held that the industrial development of mine jump give a see message. Although all the links of the industrial chain such as theoretical algorithm research, software tools, consulting service, and integrated development in the field of data mining are reflected under the promotion of universities and research institutes, the upstream market of the industrial chain is still mainly occupied by foreign enterprises [9]. Moayedi and others thought that, with the development of national information industry, especially in the recent two years in the Internet wave to promote the development of data mining industry progress rapidly. Many enterprises pay attention to the cooperation with colleges and universities, with high-end talents in related fields, pay attention to the combination of research and practicality and maximize the mining of its commercial value. It serves people's life and behavior in politics, society, culture, commerce, and health [10].

## 3. Research Method

### 3.1. Data Mining

**Figure 1:**
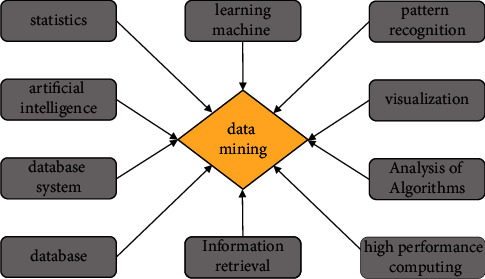
Data mining-related research fields.

Market adaptability of price prediction is not strong. Many developers in order to adapt to the changing market environment, sometimes arbitrarily lower prices or raise prices, this random pricing method is not scientific. Direct price reduction not only seriously damages the interests of e-commerce agricultural products enterprises but also is easy to reduce the goodwill of the project and lose the market. The concept of data mining broadly refers to the whole process of finding valuable patterns and knowledge in a large amount of data. This process generally includes data preparation, data preprocessing, data mining model evaluation, and presentation [11]. In a narrow sense, it only refers to a single stage of algorithm execution in the process of processing. Data mining is generally related to computer science and achieves these goals through a number of methods such as statistics, online analytical processing, intelligence retrieval, machine learning, expert systems (relying on past rules of thumb), and pattern recognition. The problem of big data has been discussed by more and more people. Massive data is stored in Internet databases or other data storage devices in various fields related to our daily life, such as business, social sciences, engineering, and medical treatment. The surge of available data is the result of the rapid development of software and hardware in a highly information society, and the data collection capacity and storage devices are increasing [12]. Generally, the pricing methods of e-commerce agricultural products marketing projects mainly include market comparison pricing method, cost pricing method, market competition pricing method, and future income pricing method. However, three factors usually need to be considered in the e-commerce agricultural products pricing practice: one is cost, including land cost, network cost, tax, and other expenses; the second is competition, the total market supply and demand, and the price of direct and indirect competitors; the third is the consumer, which is the price the target consumer can accept. Therefore, the above pricing method should be used comprehensively. In the business world, for example, huge amounts of data are generated every day, including sales records, stock transactions, product descriptions, promotions, company profits, and user feedback: Medical and health organizations have access to vast amounts of data such as medical records, patient monitoring, and medical images. Network media has become a continuous data source, such as constantly uploaded images, videos, and text information. The data obtained in these fields is almost endless. The wide application of these explosive growth data makes human life enter the data age [13]. Data mining is driven by the urgent need for powerful and comprehensive tools to automatically discover and analyze this data into regular knowledge for human use. In recent years, data mining has attracted great attention in the information industry, the main reason is that there is a large amount of data, which can be widely used, and there is an urgent need to convert these data into useful information and knowledge. The information and knowledge acquired can be used in a wide variety of applications, including business management, production control, market analysis, engineering design, and scientific exploration. Data mining is a young and developing subject, which will certainly help us develop rapidly from the data age to the real information age. Data mining is a brand new discipline, and as an application-driven research field, data mining also belongs to an interdisciplinary discipline, database system, artificial intelligence, as shown in Figure 1. Data mining is an interdisciplinary research of great significance for the successful development and wide application of data mining [14]. In this regard, e-commerce enterprises should combine the psychological characteristics of consumer groups, formulate scientific and reasonable price strategies, stimulate consumers' consumption desire, drive consumption behavior, and improve economic benefits. Different consumer groups have different needs, even if the product is the same, it is very likely to have different preferences. The reason is that the product brings different experience effects to consumers, and the degree of consumers to accept the product price and the experience effect is closely related.

**Figure 2:**
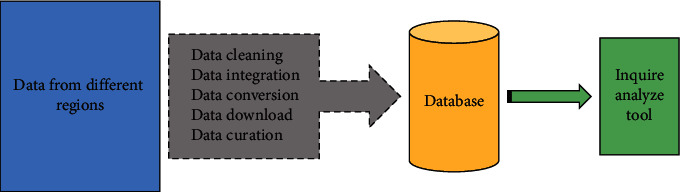
Typical working framework for data warehouse.

As a general technology, data mining can be applied to almost any field, as long as the data in these application fields has practical significance. The most basic application areas are data stored in databases, data in data warehouses, and transactional data. It consists of a set of relational data and the software that accesses and manages that data [15]. Software programs are responsible for defining data structure data storage concurrent and sharing distributed data access information consistent and secure authorization operations. The resources in the database can be fully managed, and a kind of control about the data can be realized; the utility program of the database can make the database established on a relatively complete basis, and the database can be maintained under a relatively complete database system. Benefit is always the first driving force of marketing. The project is unsalable. Generally speaking, marketers should avoid direct price reduction and can adopt a variety of disguised price strategies according to the initial pricing to deal with the consumer psychology of buying out or not buying out. For example, we can increase the number of marketing, increase quality services, or take other measures such as commodity surcharges to attract customers and enhance customer confidence. Relational database is the most widely used database. It is a collection of data tables. Each data table contains multiple attribute columns, which can store a large number of records. Each record is identified by a unique primary key. Data stored in a relational database can be manipulated with relational query statements. Query statements can carry out specific data set operations. Given a query, query statements can be converted into connection selection projection and other relational operations to obtain user demand information [16]. Relational operations can only carry out basic operations. We can conduct in-depth data pattern analysis through data mining technology.  Figure 2 is the typical working framework of data warehouse. However, when databases are located in different geographical locations, the analysis of the data described above becomes very difficult. Data warehouse can store data from different data sources to a unified location through set operations. Given a query, query statements can be converted into connection selection projection and other relational operations to obtain user demand [17].

**Figure 3:**
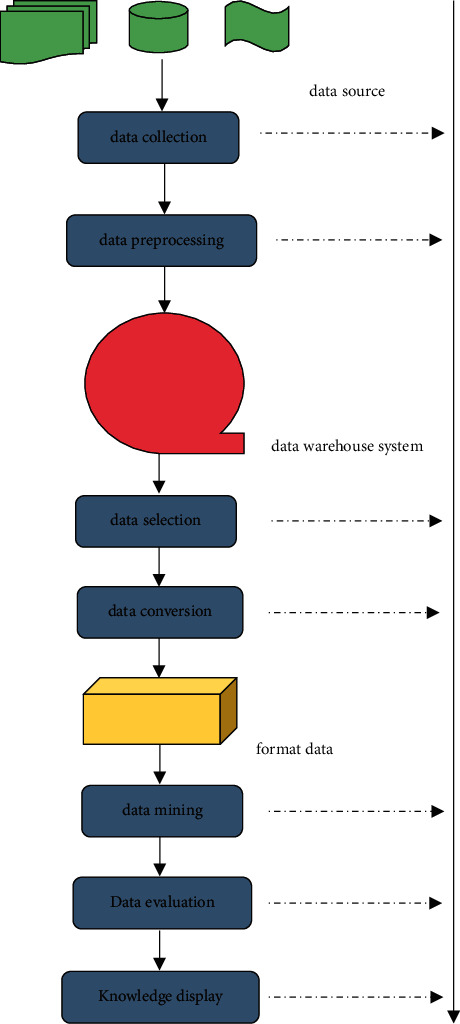
Data mining processing flow chart.

Data mining visualization can efficiently and clearly show data through graphics and images, so it has a very wide range of applications. The relationship between data can be intuitively discovered through visualization analysis technology, especially in the case of small amount of data, after data visualization processing. In the process of data cleaning, noise data can be effectively eliminated. In the process of algorithm execution, especially in the process of supervised learning, the algorithm execution process can be displayed, and human-computer interaction interface can be provided. In the final knowledge display, results can be clearly displayed. Visualization techniques can be used throughout the data mining process [18]. The process of data mining is processed according to the steps of data collection, data preprocessing, other relational operations to obtain user demand mode evaluation and knowledge display, accompanied by visual analysis technology, as shown in Figure 3.

Data collection is the process of collecting data from various data sources, such as database systems, file systems, Internet resources, multimedia data [19]. Consumers have different willingness to pay, and some people think that the product symbolizes status, regardless, the price level will choose to buy. In this regard, e-commerce enterprises should quickly adjust their pricing strategies, make accurate judgment on consumers' product demand and purchasing power by analyzing big data, and implement the differentiated pricing strategy to meet the individual needs of consumers and realize the maximization of the interest of enterprises within the price elasticity range. Data preprocessing includes two steps: data cleaning and data integration. Data cleaning can be used to remove noise from data and correct inconsistencies. Data integration is combining data from multiple data sources into a consistent data store, such as a data warehouse. Data selection is the process of selecting the data related to the analysis task in the data twarehouse system, while data transformation is the process of converting the data after data selection processing into the executable formatted data of data mining algorithm, such as the selection of feature vector of data normalization processing. Secondly, on the basis of users and professional knowledge, such as the implementation of data mining algorithms, the results of general algorithms are not necessarily meaning and value models. Model evaluation needs to determine the accuracy and effectiveness of the model. The final evaluation is a useful model, especially the data visualization analysis technology can be applied to show the whole process of data mining [20]. A time series is a sequence arranged in chronological order, usually used to mark the value of a continuous point in time with uniform time interval. Agricultural product price forecasting model is also a typical application of time series. Time series is a kind of natural sequence, so the prediction model based on the data of time series format can predict the future value through the existing observation value, and the application of this model is called regression analysis. Time series is often represented by linear graph in visualization analysis of data mining. A simple model of time series data can be represented in two ways, namely, additive model (additive) and multiplicative model (multiplicative). Daily observations of agricultural price data can be labeled as discrete time series sampled at fixed time intervals [21]. The recorded values *x*_1_, *x*_2_, *x*_3_,…*x*_*n*_ obtained in the discrete time *X* series of agricultural product price at time *t*_1_, *t*_2_, *t*_3_,…*t*_*n*_ are written as

(1) $$\begin{array}{c} X=\left(x_{1},x_{2},x_{3},\ldots ,x_{n}\right);x_{i}\in R_{0}. \end{array}$$

In this way, the prediction of agricultural product price data based on time series can be defined as (*X*, *X*′), namely, the next group of values of the same time interval using *X* time series. In the application, the interval can be defined as 1 day, which means that the price observations of *n* consecutive days are used to predict the price values of several (*n* + 1) days later. There are many prediction models of time series, such as ARMA model, which is widely used in statistics, and classical algorithms in data mining, such as neural network algorithm, Bayesian algorithm, fuzzy set algorithm, support vector machine, and nearest neighbor algorithm [22]. It is based on the classical KNN algorithm and BPNN algorithm time series prediction model, and according to the specific application of the prediction model, an improved algorithm and an optimized model are proposed.

### 3.2. Classical KNN Algorithm

K-nearest neighbor algorithm (KNN), in order to distinguish it from the improved KNN algorithm, it is called the classical KNN algorithm. Classical KNN algorithm is a learning algorithm based on analogy, that is, the algorithm of prediction between prediction tuples and training tuples [23]. Training tuples are described by attribute values, and there is a known social effect majoring, so that each training tuple is like a function of dimensional space, as follows:

(2) $$\begin{array}{c} f\left(X\right)=f\left(x_{1},x_{2},\ldots ,x_{n}\right). \end{array}$$

*x*_1_, *x*_2_,…*x*_*n*_ represent the attribute value, and *f*(*X*) represents the classification label. Query statements can carry out specific data set operations. Given a query, query statements can be converted into connection selection projection and other relational operations to obtain user demand KNN in the pattern space, and this *k* tuple is the K-nearest neighbor of the unknown tuple. The similarity is calculated by the Euclidean distance formula, as follows:

(3) $$\begin{array}{c} dist\left(X_{1},X_{2}\right)=\sqrt{\sum_{i=1}^{n}{\left(x_{1i}-x_{2i}\right)}^{2}}. \end{array}$$

The classical KNN algorithm can be used for numerical prediction, that is, to return the true predicted value according to an unknown tuple. The price marketing strategy must grasp the objective basis of pricing; conduct market research; objectively analyze the national policy, the market environment, the value of the development project itself, the supply and demand relationship of the real estate market, the market competition situation, the psychology of the home buyers, and other factors; choose the scientific pricing method; and adopt the corresponding strategies.

### 3.3. BPNN Algorithm

Artificial neural networks (ANNs) refers to an algorithmic structural model that mimics the behavior of animal neural features by interconnecting internal nodes. In the learning process of the neural network, through the training of training tuples, the weight value is constantly adjusted and finally evolves into a complete neural network structure for information processing. Although the interpretability of neural network algorithm is poor, its high accuracy with high tolerance of noisy data and in the case of known data volume makes it widely used in various forecasting models. The back propagation neural network (BPNN) algorithm proposed by scientists in 1986 is the most widely used neural network algorithm [24]. According to the trained prediction model based on the BPN algorithm, we can directly get our expected predicted value as long as we input the tuple to be predicted. E-commerce agricultural products must be combined with the relevant actual situation, affect the e-commerce precision marketing strategy, and adopt a scientific and reasonable way to improve the precision marketing strategy and to maximize the growing and changing consumer needs of users in the background of big data. We will promote the rapid and stable development of e-commerce enterprises.

### 3.4. Improved KNN Algorithm

The classical KNN algorithm has the following two defects. First, the education formal is used to measure the development, and the same weight is assigned to each attribute value. Therefore, the prediction accuracy will be seriously affected when there is noise data. Even if weight is assigned to attribute values, the determination of weight is still a problem to be solved because of the multidimensional characteristics of prediction sequence. Secondly, the selection of value can only be determined through experiments. Generally, it starts from 1 and evaluates the prediction error of training tuples. As the *k* value changes, the value that minimizes the training error will be selected for constructing the prediction model because the time complexity is relatively high and user guidance is needed. Aiming at the two defects of classical KNN query statements can carry out social data set operations [25]. In order to improve the prediction accuracy and reduce the time complexity, it also solves the problem of weight allocation and *k* value selection. Because the improved KNN method mainly depends on limited surrounding samples, rather than the method of distinguishing the class domain to determine its category, KNN method is better than other methods for the sample sets to be classified that overlap more in the class domain. High open low marketing method gives people the embodiment of high quality of the project, but it is difficult to gather popularity, and there is a certain marketing risk. High opening and low walking tactics are suitable for the following e-commerce products: projects with innovative and unique selling points; projects with good comprehensive performance and slow functional depreciation. Against the first defect of the classic KNN algorithm, because the prices for agricultural products have the characteristics of time sequence, when to predict the price of agricultural products, the role of the attribute value showed a trend of increase, meaning that its weight is increasing, the corresponding attribute value increasing, and this relationship is not linear increase, the binomial function with similar characteristics.

**Figure 4:**
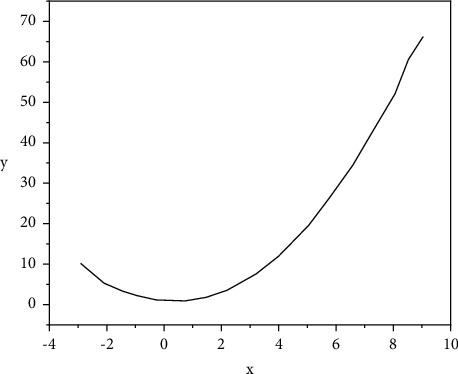
Graph of a binomial function.

As shown in Figure 4, on the right side of the axis of symmetry, as the abscissa value increases, the corresponding ordinate value also increases, and the change rate (slope) is positive. At this point, the attribute value of the prediction sequence (*x*_1_, *x*_2_,…*x*_*n*_) is regarded as the abscissa value, and the weight value is regarded as the ordinate value, and then the first improvement of the improved KNN algorithm is obtained, namely, the binomial function Euclidean distance formula, namely,

(4) $$\begin{array}{c} \mathrm{dist}\left(X_{1},X_{2}\right)=\sqrt{\sum_{i=1}^{n}\left(ai^{2}+bi+c\right){\left(x_{1i}-x_{2i}\right)}^{2}}. \end{array}$$

The variable *a*, *b*, and *c* in the formula is the binomial coefficient, and the variable *i* represents the attribute value dimension of the prediction project so that the weight problem of a specific data set operations. Given a query, query statements can be converted into connection selection projection and other relational operations to obtain user demand. For the second defect, we combine the determination of value *k* with the determination of binomial function coefficient *a*, *b*, and *c* and use parameter optimization algorithm to determine.

### 3.5. PSO Algorithm Optimization of Parameters

**Figure 5:**
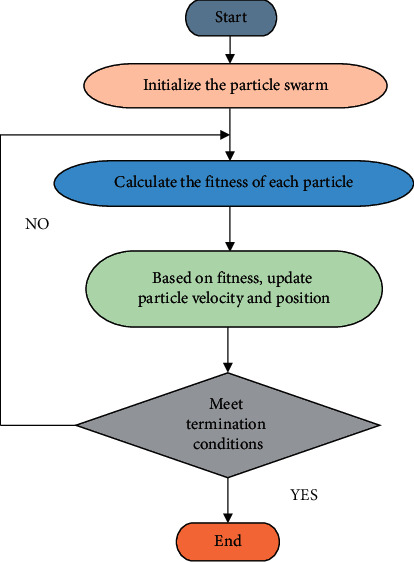
Basic flow chart of the PSO algorithm.

Particle swarm optimization algorithm (PSO) is an evolutionary algorithm, an optimization algorithm proposed by American scientists in 1995 by simulating the process of achieving social behavior goals (such as group foraging and avoiding natural enemies) through the cooperation between individual behaviors of birds or fish groups. Particle swarm optimization algorithm is a heuristic algorithm. It first initializes the parameters to be optimized into a group of random solutions, then finds the optimal solution through iteration, and evaluates the quality of the solution through adaptive function in the iterative process. The algorithm can be solved in a wide range of candidate sets with little or no assumption of solution. The PSO algorithm has the advantages of easy implementation, high precision, fast convergence, and simplicity. It does not require the optimized function to have properties such as differentiability, derivation, and continuity. The convergence speed is fast, the algorithm is simple, and it is easy to program and implement, making it widely used. Various examples applied to parameter optimization. The PSO algorithm can be well applied to the parameter optimization of BPNN algorithm and improved KNN algorithm. When PSO algorithm is used to optimize the parameters of the BPNN algorithm, the topological structure of neural network model should be defined according to the specific application of the algorithm.  Figure 5 is the basic flow of PSO algorithm.

**Figure 6:**
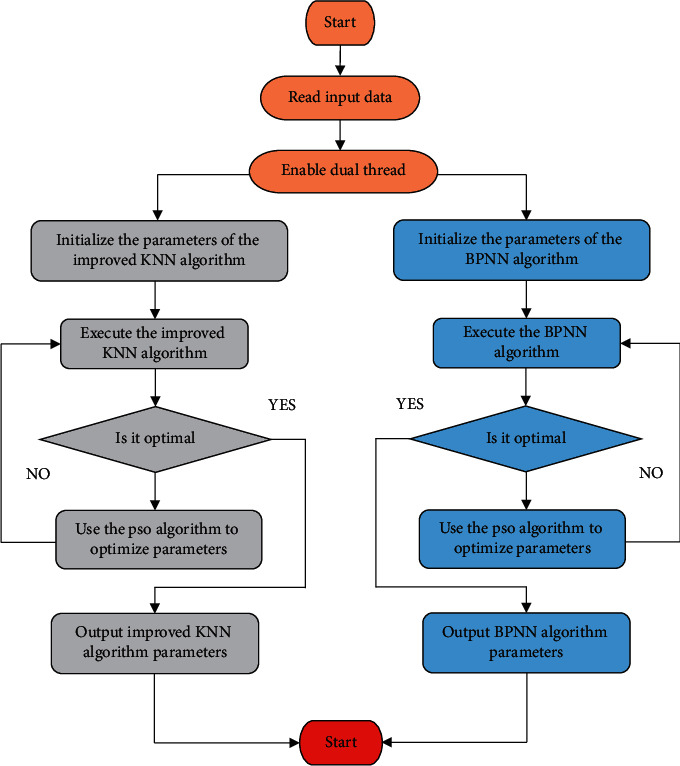
Process flow chart of parameter optimization of the prediction model.

As shown in Figure 6, the parameters to be optimized of the BPNN algorithm and the improved KNN algorithm are randomly initialized into the positions and velocities of a group of particles. Then, the fitness of each particle is calculated according to the fitness function. According to the specific application of, the fitness function is defined as

(5) $$\begin{array}{c} E=\frac{1}{N}\sum_{i=1}^{N}{\left(T_{i}-y_{i}\right)}^{2}. \end{array}$$

Value *N* is the number of training samples, *T* and *y*, respectively, represent the real value of each sample in the training sample set and the actual value of training output. Value *E* represents error, and the smaller the error, the higher the fitness. The fitness function determines the local optimal position of the current particle *P*_best_ and the global optimal position of the population *G*_best_, and then according to the velocity change formula, namely,

(6) $$\begin{array}{c} V_{i}=\omega \times V_{i}+c_{1}\times \mathrm{rand}\left(\right)\times \left(P_{\mathrm{best}}-p_{i}\right)+c_{2}\times \mathrm{rand}\left(\right)\times \left(G_{\mathrm{best}}-p_{i}\right). \end{array}$$

The formula is proposed by 1998 to calculate the change speed of particles, in which *ω* is the inertial weight of particles, which can control the search space of particles. A large value can ensure the search in a wide space, while a small value can ensure the convergence of parameters. In the experiment, the value is 0.75, C1 and C2 represent the cognitive ability of the particle, which can control the local optimal position *P*_best_ and *G*_best_, respectively. The value 1.25 in the experiment is a random number between 0 and 1, and *ran*  *d*() is used to randomly assign the position 0 to 1 and speed of the particle. The velocity value can be calculated according to equation (6), and then, the change formula of position can be obtained, namely,

(7) $$\begin{array}{c} p_{i}=p_{i}+v_{i}. \end{array}$$

In order to control the accuracy and convergence of the algorithm, the change interval of velocity was set as [−1,1], and the change interval of position was set as [−1,1]. Finally, when the termination condition is met, the position of the best particle is output as the best parameter value of the parameter to be optimized to end the algorithm; otherwise, the iteration cycle continues. The termination condition is set as the rate of change of the global optimal position meeting the minimum limit.

### 3.6. Data Processing

The research required to collect from data sources is in the database. Because of jump, import it into the local SQLServer database for further processing and analysis. These high quality data are stored in the local SQLServer database, but these data need to ensure the practicability and correctness of model prediction. Data cleaning strategy is to eliminate the weight through computer programming. When the wrong data is missing, the incomplete data is cleaned manually according to the time field, referring to the adjacent complete data in the context and according to the change trend of adjacent data. In order to be more intuitive to analyze and process the data in the database, the data cube technology is used for data integration. Due to the inconsistency of the quantization range of the collected data, the expansibility of the prediction model is stronger, and the convergence of the neural network algorithm is accelerated. Unified quantitative mapping between data [0, 1] is carried out, and formula (8) is the normalized formula adopted:

(8) $$\begin{array}{c} f\left(x\right)=\frac{x}{x_{\max}+y}. \end{array}$$

*x* is the unit price of the product, *x*_max_ is the historical maximum unit price of the current product in the current market collected, *y* is a user-defined variable, usually with a smaller value. *x*_max_ is 2.23, and *y* is 0.2. Through the above processing, the data can be used to perform the prediction model algorithm.

### 3.7. Establishment of the Prediction Model

In order to have a more thorough understanding of consumers' purchasing decision-making process, more and more experts and scholars begin to pay attention to the preference performance of consumers when choosing goods and their internal psychological mechanism. The rational choice theory based on the “rational economic man” hypothesis holds that the measure of the utility of a commodity is unrelated to the set of alternative choices in which the commodity is located, and in any case, consumers will choose the commodity or combination of goods that meets the maximum utility. The training samples and reference samples were taken as input data, and the improved KNN algorithm and BPNN algorithm were used to the quantization range of model. PSO algorithm is used to optimize the parameters of the improved KNN algorithm and the threshold weight of the BPNN algorithm. Finally, an optimal inconsistency of the quantization range, strong prediction stability, and low prediction time complexity is obtained. Figure 6 shows the process flow chart of parameter optimization of the prediction model.

**Figure 7:**
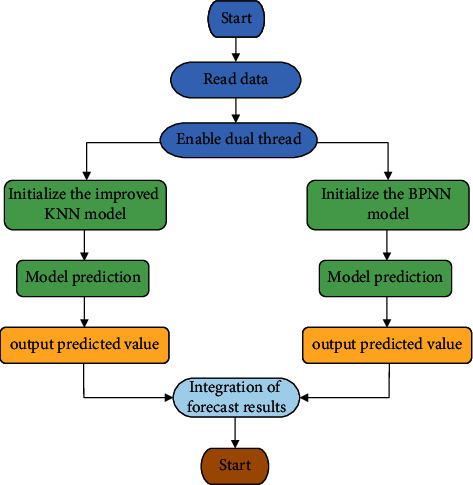
Flow chart of the agricultural product price prediction model based on the KNN-BPNN algorithm.

Input the data to be predicted, optimize the parameters, initialize the improved KNN algorithm prediction model and BPNN algorithm prediction model, respectively, and get the predicted values, and integrate the two predicted values to get the expected predicted values. When consumers choose convenience products, the utility gained from the comparison of the price and quality of alternative products is less than that of consumers' evaluation of related search costs. For consumers, convenience products have low perceived risks, and consumers' purchasing behavior is largely habitual or impulsive.  Figure 7 is the flow chart of the agricultural product price prediction model based on the KNN-BPNN algorithm:

The prediction results of the object detection algorithm should include the image, the object category in the image, and the border position of each object. Maintaining the stability of prediction accuracy while ensuring prediction accuracy is the most basic requirement and the most important goal of prediction result integration. The quality of the prediction result integration strategy plays a decisive role in this goal. Three strategies are proposed. One is to take the average value of the predicted output value of the two strategies. Strategy 2: compared with the last one-dimensional data to be predicted, the value with small change is taken as the output predicted value; Strategy 3: compared with the last one-dimensional data of the predicted input, the value with large change is taken as the predicted value of the output.

## 4. Interpretation of Result

### 4.1. Prediction Results Integration Strategy Selection

The above paper describes three strategies for the integration of prediction results. Through several experiments with test samples, the results are shown in Table 1. The prediction error of strategy 2 is the smallest, and the final prediction model chooses strategy 2. Price strategy is the most important part of the marketing portfolio. Compared with other marketing strategies, price strategy is the most difficult factor to determine in the controllable factors of enterprises, which requires enterprises to pay more attention to the price decision of commodities. Price strategy includes stage price strategy, geographic pricing strategy, and discount and concession strategy. Promotion refers to the promotion work carried out by enterprises in order to influence consumers' purchasing behavior, stimulate consumers' purchase desire, and increase the sales volume of products.

### 4.2. Prediction Model Performance Comparison

Although the classical KNN algorithm has a large prediction error, it has the strongest stability, which proves that the improved KNN algorithm has a good stability. Compared with the classical KNN algorithm, the improved KNN algorithm can not only reduce the prediction error by about 10% but also guarantee the stability of prediction, which fully proves the superiority of this algorithm. The prediction error of the BPNN inconsistency of the quantization range is about 1% lower than that, but there is a large difference in the order of stability. Finally, the prediction model based on the improved KNN algorithm and BPNN algorithm can ensure the lowest prediction error and strong stability. Under the background that the prediction accuracy is the most important objective, the validity of the prediction model is fully proved. The experimental results are the classical KNN algorithm, the improved KNN algorithm, the BPNN algorithm and the actual output of the prediction model based on the KNN and BPNN algorithm and the predicted output results. The classical KNN algorithm has the maximum shadow area, and the prediction model based on the combination of the two algorithms has the minimum shadow area, that is, the minimum prediction error. As for the computational efficiency, since the database data is updated in the unit of day, it has low requirements in the specific application. However, from the perspective of algorithm, using the PSO algorithm to optimize parameters can achieve fast convergence and effectively reduce the running time. The improved KNN algorithm is used to reduce the number of parameters that need to be optimized to meet the needs of linear optimization. The parameters of the improved KNN algorithm and BPNN algorithm are optimized by two threads, respectively. The running optimization time is kept as the running time of optimizing BPNN algorithm parameters, which ensures the optimal model implementation.  Table 2 shows the running time of the agricultural product price prediction model constructed by the four algorithms, in milliseconds (ms).

As shown in the above table, the time efficiency of the improved KNN algorithm is at least three times higher than the execution efficiency of the BPNN algorithm in the experiments, which also demonstrates the feasibility of KNN-BPNN algorithm in dual-thread execution. Then, the improved KNN algorithm is used to predict consumers' intention to buy agricultural products, and the accuracy is verified with different threshold values *K*, as shown in Table 3.

In the process of establishing the above model, the calculation method of the KNN algorithm not only correctly predicted the price of agricultural products but also carried out corresponding prediction analysis of consumers' intention to buy agricultural products, with the highest accuracy of 94.2%. At the same time, it also provides a good data basis for scheme design in the future precision marketing process, which can provide accurate message push to target users. The specific representation of a price strategy uses a 5-level Likert scale (never, occasionally, sometimes, often, and always) approach. The results showed that the average frequency of the individual sample enjoying freight subsidy benefits was “sometimes,” while the average frequency of enjoying noncumulative quantity discounts was only “occasional.” Therefore, businesses should increase the frequency of launching price marketing concessions, especially for convenience products, through the guidance of price strategy, so as to increase the total purchase amount of consumers and then increase the total income of e-commerce agricultural products themselves.

## 5. Conclusion

This paper studies the prediction inconsistency of the quantitative range of the model and uses the data mining algorithm to establish the e-commerce agricultural product price and purchase intention prediction model with high prediction accuracy, strong prediction stability, and low computational complexity. In the network environment, marketing means are diverse, and marketing forms are gathered. Specific marketing methods will have a significant impact on the compromise effect, and the compromise effect shown in consumers' purchase decisions needs to be further studied in detail. Secondly, for the alternative commodity set, whether the price difference between extreme options and discount options and the discount intensity in the price strategy will also affect the discount effect. On the basis of classical nearest neighbor algorithm, this paper combines binomial function with Euclidean distance formula when calculating nearest neighbor by similarity. Particle swarm optimization is used to optimize the parameters of binomial function coefficient and K value of nearest neighbor algorithm, and an improved nearest neighbor algorithm based on binomial function to calculate the weighted Euclidean distance is proposed. The forward feedback neural network algorithm is widely used in various prediction models. Aiming at the advantages of strong prediction stability of the improved nearest neighbor algorithm and high prediction accuracy of the improved forward feedback neural network algorithm, this paper effectively combines the two improved methods to establish the best prediction model for the application of e-commerce agricultural product price prediction. It is expected that this will play a guiding role in the production and sales of e-commerce agricultural products. The results are as follows: (1) a series of evaluation of the inconsistency of the quantization range model based on the KNN-BPNN algorithm was carried out from the accuracy stability and computational efficiency of the prediction model. By comparing with the BPNN algorithm optimized by the PSO algorithm and KNN algorithm improved by the classical KNN algorithm, the experiment proves that the inconsistency of the quantization range of model based on the KNN-BPNN algorithm has the highest prediction accuracy. (2) Based on the excellent performance of the classical KNN algorithm in stability, the stability of inconsistency of the quantization range of model established based on the KNN-BPNN algorithm also has a good performance. In terms of time efficiency, since the agricultural inconsistency of the quantization range model established based on the KNN-BPNN algorithm needs to optimize the parameters of the two algorithms, at least the time of parameter optimization of the BPNN algorithm is required even in the case of dual threads, but for the specific application, its importance is relatively low. In conclusion, the above experiments prove the feasibility and effectiveness of the e-commerce agricultural product price prediction and purchase intention model based on the KNN-BPN algorithm. The overall trend of the development of data mining technology has gradually changed from dealing with simple mining problems to solving complex mining problems. Before that, there were many theoretical studies on data mining in China, but there were few studies on data mining application systems and algorithm testing. The same data set used in relevant international research can be used to compare and test the existing algorithm and the new improved algorithm, so as to test the rationality of the new algorithm and effectively strengthen the research on the effectiveness of mining results. In this regard, e-commerce enterprises should comprehensively consider the market development trend and the actual development of their own business, seriously explore the precise marketing strategy of e-commerce under big data, and pave the way for achieving more significant benefit goals.

## Figures and Tables

**Table 1:** Prediction result integration strategy prediction error.

The serial number	Strategy one prediction error	Strategy two prediction error	Strategy three prediction error
1	0.191002	0.175049	0.222563
2	0.190227	0.177673	0.230734
3	0.215551	0.198954	0.256976
4	0.207145	0.194594	0.228272
5	0.192178	0.183855	0.227506
6	0.208932	0.206051	0.218532
7	0.195562	0.185473	0.234474
8	0.200241	0.205375	0.234389
9	0.221113	0.254049	0.381148
10	0.289714	0.252895	0.381102
Mean	0.211167	0.203397	0.246570

**Table 2:** Comprehensive comparison table of model running time.

The serial number	Classical KNN algorithm	Improved KNN algorithm	BPNN algorithm	KNN-BPNN algorithm
1	16	1590	5124	5124
2	34	1688	6004	6004
3	25	1745	2061	2061
4	23	1522	6024	6024
5	14	1451	5714	5714
6	16	1537	7970	7970
7	33	1512	5815	5815
8	6	1520	6549	6549
9	43	1482	5741	5741
10	19	1551	4923	4923
11	10	1487	4748	4748
12	13	1512	3757	3757
13	16	1548	5041	5041
14	32	1539	3163	3163
15	29	1454	4845	4845
16	25	1467	6852	6852
17	26	1498	4747	4747
18	30	1535	3101	3101
19	7	1523	6304	6304
20	10	1526	2766	2766
Average	21	1534	5062	5062

**Table 3:** Prediction accuracy of different threshold *K*.t

Threshold value	5	7	10	12
Precision	92%	91.4%	94.2%	91.1%

## Data Availability

The data that support the findings of this study are available from the corresponding author upon reasonable request.
